# Diagnostic performance of coronary computed tomography angiography stenosis score for coronary stenosis

**DOI:** 10.1186/s12880-024-01213-8

**Published:** 2024-02-09

**Authors:** Qing-feng Xiong, Xiao-rong Fu, Lei-zhi Ku, Di Zhou, Sheng-peng Guo, Wen-sheng Zhang

**Affiliations:** 1 Hainan Enhance International Medical Center, Boao, China; 2 Wuchang Hospital of Wuhan University of Science and Technology, Wuhan, China; 3 Wuhan Asia Heart Hospital of Wuhan University of Science and Technology, Wuhan, China

**Keywords:** Coronary angiography, Coronary artery disease, Coronary computed tomographic angiography, Stenosis score, Diagnosis performance

## Abstract

**Background:**

Coronary computed tomography angiography stenosis score (CCTA-SS) is a proposed diagnosis score that considers the plaque characteristics, myocardial function, and the diameter reduction rate of the lesions. This study aimed to evaluate the diagnostic performance of the CCTA-SS in seeking coronary artery disease (CAD).

**Methods:**

The 228 patients with suspected CAD who underwent CCTA and invasive coronary angiography (ICA) procedures were under examination. The diagnostic performance was evaluated with the receiver operating curve (ROC) for CCTA-SS in detecting CAD (defined as a diameter reduction of ≥ 50%) and severe CAD (defined as a diameter reduction of ≥ 70%).

**Results:**

The area under ROC (AUC) of CCTA-SS was 0.909 (95% CI: 0.864–0.943), which was significantly higher than that of CCTA (AUC: 0.826; 95% CI: 0.771–0.873; *P* = 0.0352) in diagnosing of CAD with a threshold of 50%. The optimal cutoff point of CCTA-SS was 51% with a sensitivity of 90.66%, specificity of 95.65%, positive predictive value of 98.80%, negative predictive value of 72.13%, and accuracy of 91.67%, whereas the optimal cutoff point of CCTA was 55%, and the corresponding values were 87.36%, 93.48%, 98.15%, 65.15%, and 88.60%, respectively. With a threshold of 70%, the performance of CCTA-SS with an AUC of 0.927 (95% CI: 0.885–0.957) was significantly higher than that of CCTA with an AUC of 0.521 (95% CI: 0.454–0.587) (*P* < 0.0001).

**Conclusions:**

CCTA-SS significantly improved the diagnostic accuracy of coronary stenosis, including CAD and severe CAD, compared with CCTA.

**Supplementary Information:**

The online version contains supplementary material available at 10.1186/s12880-024-01213-8.

## Background

Coronary artery disease (CAD) is a well-documented risk factor for mortality and morbidity of cardiovascular diseases among adults [1]. Chest pain is the most common symptom of CAD; however, many CAD cases occur asymptomatically without warning [2]. Invasive coronary angiography (ICA) is the standard diagnostic method of CAD in clinical practice. By illustrating the site, extent, and severity of coronary stenosis, ICA allows us to determine the necessity of immediate interventional procedures.

Considering the potential adverse effects of ICA, several noninvasive imaging technologies have been developed in practice [3]. Coronary computed tomography angiography (CCTA), a valuable diagnostic approach [4, 5], is recommended as a primary measurement method to distinguish between patients with low or intermediate risk of CAD [6, 7]; however, its specificity in determining the functional significance of coronary stenosis is limited. For this reason, adenosine-stress dynamic CT myocardial perfusion imaging and the noninvasive prediction of fractional flow reserve (FFR) techniques have gradually assessed and improved the diagnostic accuracy of coronary stenosis [8, 9]. CT-derived FFR (CT-FFR) combining CT and mimetic FFR techniques [10] provides more efficient noninvasive functional imaging to confirm ischemia in patients with moderate or suspected coronary stenosis [11]. However, calcified plaques, heart rate, and arrhythmia are major confounders for CAD diagnosis by using CT-FFR.

For patients with severe coronary artery stenosis, myocardial ischemia often merges with segmental myocardial dysfunction. Moreover, myocardial perfusion is also affected by coronary artery stenosis [12]. Therefore, integrating information on coronary artery stenosis, coronary artery plaque, and myocardial function is a vital step before the diagnosis [13–15].

CCTA-stenosis score (CCTA-SS), a novel diagnosis approach developed by our group [16], combines the data on diameter stenosis, lesion features, first-pass myocardial perfusion, and myocardial function through a comprehensive evaluation to eliminate the disturbance caused by calcified plaques, heart rate, and arrhythmia and to improve the diagnostic accuracy of coronary stenosis. The details of the scoring method have appeared in our previous article [16]. In the present study, we enrolled patients who planned to undergo CCTA and ICA assessments and compared the diagnostic performance of CCTA-SS with CCTA for CAD and severe CAD.

## Methods

### Study design and settings

This retrospective study collected 228 patients who underwent CCTA and ICA, to assess the diagnosis performance for coronary artery lesions at the Wuhan Asia Heart Hospital from May 2016 to October 2016. Patients’ records were reviewed and extracted. The time interval between CCTA and ICA measurements was less than 1 week. Both CCTA and CCTA-SS were used to evaluate the stenosis, using ICA as the gold standard.

The study followed the Declaration of Helsinki and was approved by Institutional Review Board of the Wuhan Asia Heart Hospital Affiliated to Wuhan University of Science and Technology (2,017,016), and the need for informed consent was waived.

### Patients

Patients who met any one of the following criteria would be enrolled. The assessments were as below: (1) atypical chest pain symptoms; (2) borderline severe stenosis; the lesion range and stenosis severity needed to be assessed before clinical treatment; (3) presenting with typical angina with a Global Registry of Acute Coronary Events (GRACE) score of low-to-moderate risk (< 140); and (4) at least one visible LAD lesion. Patients with the following conditions were excluded: aged ≤ 20 years; kidney failure; intolerant to the iodine contrast agents; obstructive pulmonary disease; atrial fibrillation; a left ventricular ejection fraction of ≤ 35%; ST-segment elevation of acute myocardial infarction; left ventricular aneurysm; history of revascularization; ventricular arrhythmia; spastic bronchial asthma; cardiomyopathy or II–III degrees of atrioventricular block; and small vessel diameter (< 2 mm) or deep or long myocardial bridges (> 30 mm).

### CCTA examination

All patients underwent the dual-source CT protocol (Somatom Definition Flash; Siemens Healthcare, Forchheim, Germany) with the prospective scanning technology by professional radiologists according to the standard operating procedure [17]. Briefly, all patients were given 0.8 mg glycerol trinitrate sublingually immediately before the scan. To achieve the target heart rate of < 85 beats/min, 5–15 mg atenolol was administered orally. A test bolus approach made sure the contrast agent transit time. CT angiography was then performed with an injection of 50–65 mL contrast agent with a flow velocity of 4.3-5.0 mL/s, followed by 30 mL saline at the same flow velocity. The maximum intensity projection (MIP) was rendered, in addition to the axial and oblique multi-planar reconstructions (MPR).

The coronary centerlines were performed and semiautomatically analyzed using a commercially available workstation (Extended Brilliance Post Processing Workspace, Philips Medical Solutions, Netherlands), including the stenosis degree of lesions. Two senior imaging experts with more than five years of experience in CCTA interpretation, who were unaware of the ICA results, independently performed the assessments, and reach a consensus by consulting. The plaques were divided into four categories: non-calcified lipid, non-calcified fiber, calcified, and mixed. High-risk plaques were defined as lipid-rich with spot calcification (CT < 30 HU) and a vascular remodeling index of more than 1.10 [18, 19].

The quantification index of luminal narrowing and vessel remodeling in the target lesion did well using dedicated software by two experienced observers. The index included lumen diameters and segment lengths of the lesions utilizing MPR images, plaque features were observed on MIP and MRP images, the myocardial perfusion was observed on the short axis of the left ventricle during diastole phase, and the myocardial function evaluation needed to combine systolic and diastolic phases. The calculation formula is as follows: (relative normal lumen diameter near the lesion − lumen diameter at the lesion) × 100 /relative normal lumen diameter near the lesion. We categorized the stenosis severities into three levels: <50%, 50–70%, or ≥ 70%.

A difference in the CT values of the anterior and lateral walls of the left ventricle at the level of the papillary muscles of the short axis exceeding 20 HU was defined as an abnormity in the early myocardial perfusion [20]. The myocardium thickening rate was calculated based on the measurements of the thickness of the myocardium during the diastolic period (mitral valve is fully open) and systolic period (mitral valve is completely closed) [13].

### CCTA-stenosis score (CTCA-SS)

The efficacy of CCTA-SS was evaluated and validated in our previous study [16]. In addition to the degree of stenosis and myocardial function, the scoring system considered plaque characteristics. Previous evidence also suggested that the precision of CAD prediction significantly improved after including data regarding plaque characteristics and stenosis severity [21]. A total of seven features were included in the scoring system: diffusion conditions, centrality, level of risk, the diameter of stenosis based on the segment involvement score [22], early perfusion status, myocardial thickness, and thickening rate. Weighted scores for these features were assigned according to the logistic regression model analysis and professional experiences [16]. The total CCTA-SS was 111; It was calculated by two highly experienced physicians with ten years of CCTA image diagnosis experience, respectively, who reach a consensus through consultation. A higher score indicated a higher risk of CAD. The detailed scoring method is presented in Additional Table S1.

### Invasive coronary angiography (ICA)

Invasive coronary angiography was performed as per the standard operating procedure that was used as the golden standard method for the diagnosis of CAD and severe CAD. At least six views of the left coronary arteries and two views of the right coronary arteries were projected. Two cardiologists with over ten years of experience in cardiac catheterization, who were unaware of the results of CCTA, independently interpreted the angiograms. All coronary artery segments ≥ 1.5 mm in diameter were visually and quantitatively analyzed comparison with data from CCTA. Quantitative coronary angiography for most severe stenosis was performed. A stenosis of ≥ 50% was defined as CAD, and that of ≥ 70% was defined as severe CAD [23].

### Other data collection

Demographic information, clinical characteristics, and treatment records within the 6-month follow-up period of all patients were extracted from their medical records.

### Statistical analyses

Means ± standard deviations were calculated for the continuous variables, and frequency (%) was calculated for categorical variables. The receiver operating characteristic (ROC) curve and areas under the curve (AUC) were calculated to compare the diagnostic performance of CCTA and CCTA-SS for both CAD and severe CAD. All statistical analyses were performed using SPSS 22.0 (IBM Corp. Armonk, NY, USA). A two-tailed *p-*value of < 0.05 was considered statistically significant.

## Results

### Baseline characteristics

A total of 228 patients (*n* = 89, 39% females) aged 59.10 **±** 9.9 years were included in the study (Table 1). The time interval between CCTA and ICA examinations was not more than one week for all participants, with a median of 4 days (range, 2–7 days). Of these, 82.5% of the patients had angina, 42.1% had high -risk plaques, 58.3% had ST-T segmental changes, and 71.1% had cute coronary syndrome. Moreover, 27.6% of the patients were classified into Kiliip II degree, and 28.9% were diagnosed with medium risk of GRACE score. There were no one with high risk for GRACE. Most the patients underwent drug treatment (70.6%), and 29.4% received percutaneous coronary intervention or CABG.

**Table 1:** Characteristics of 228 CAD patients underwent CCTA and invasive coronary angiography (ICA) examination

Clinical characteristics	Mean ± SD/ n (%)
Total number	228
Female, n (%)	89(39%)
Age (years), mean ± SD	59.10 **±** 9.938
Smoking, *n* (%)	56(24.6%)
Hypertension, *n* (%)	91(39.9%)
High blood lipids, *n* (%)	154(67.5%)
Positive for troponin, *n* (%)	42(18.4%)
Average heart rate (times/minute), mean ± SD	73.5 **±** 12.37
Average blood creatinine, mean ± SD	70.1 **±** 19.20
Abnormal sugar tolerance, *n* (%)	43(18.9%)
Angina, *n* (%)	188(82.5%)
Stable	26(11.4%)
Unstable angina	154(67.5%)
NSTEMI	8(3.5%)
High-risk plaques, *n* (%)	96(42.1%)
Kiliip, *n* (%)	
I	165(72.4%)
II	63(27.6%)
Grace Score, *n* (%)	
Low risk	162(71.1%)
Medium risk	66(28.9%)
ECG, *n* (%)	
ST-T segmental changes	133(58.3%)
Normal	95(41.7%)
Clinical intervention, *n* (%)	
Drug treatment	161(70.6%)
PCI & CABG	67(29.4%)
Acute coronary syndrome, *n* (%)	
No	66(28.9%)
Yes	162(71.1%)

ECG: Electrocardiogram; PCI: Percutaneous Coronary Intervention; CABG: Coronary Artery Bypass Grafting; NSTEMI: Non-ST Elevation Myocardial Infarction

### Diagnostic accuracy of CCTA and CCTA-SS

**Fig. 1:**
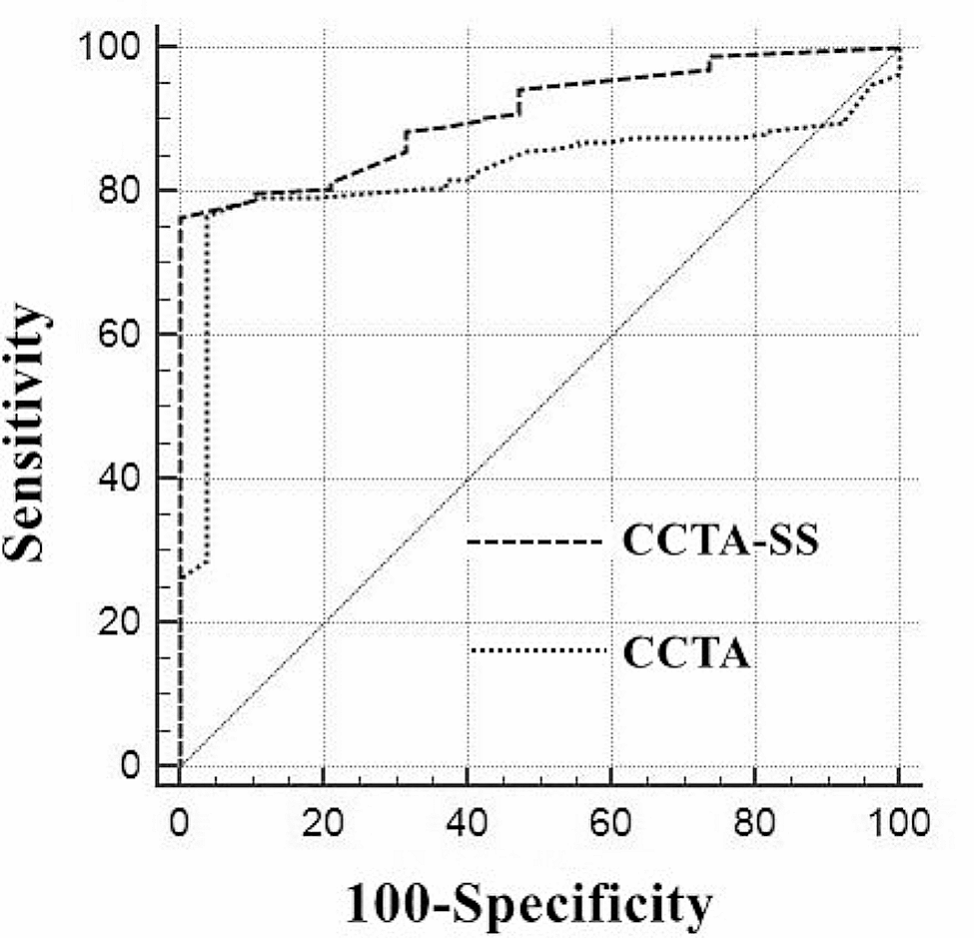
Receiver operating curve of coronary computed tomography angiography and coronary computed tomography angiography stenosis score for coronary artery disease diagnosis

For diagnosis of CAD, the AUC was 0.909 (95% CI: 0.864–0.943) for CCTA-SS and 0.826 (95% CI: 0.717–0.873) for CCTA, respectively (*P* = 0.0352) (Fig. 1). At the optimal cutoff point of 55% for CCTA and 51% for CCTA-SS, the sensitivity (CCTA-SS vs. CCTA: 90.66% vs. 87.36%), specificity (95.65% vs. 93.48%), positive predictive value (98.80% vs. 98.15%), negative predictive value (72.13% vs. 65.15%) and accuracy (91.67% vs. 88.60%), there was significant difference between CCTA-SS and CCTA (Table 2; Fig. 1).

**Table 2:** Performance of CCTA and CCTA-SS for diagnosing CAD

	Optimal threshold	CAD by ICA		Specificity	Sensitivity	NPV	PPV	Accuracy	AUC (95% CI)
		No	Yes						
**CAD diagnosis: ICA ≥ 50%** ***n*** ** = 228**									
CCTA	55%								
No		43	23	93.48%	87.36%	65.15%	98.15%	88.60%	0.826 (0.771～0.873)
Yes		3	159						
CCTA-SS	51%								
No		44	17	95.65%	90.66%	72.13%	98.80%	91.67%	0.909 (0.864～0.943)
Yes		2	165						
**P-values**									<0.05
**Severe CAD diagnosis: ICA ≥ 70%** ***n*** ** = 228**									
CCTA	68%								
No		50	33	54.95%	75.91%	60.24%	71.72%	67.54%	0.521 (0.454～0.587)
Yes		41	104						
CCTA-SS	51%								
No		89	10	97.80%	92.70%	89.90%	98.45%	94.74%	0.927 (0.885～0.957)
Yes		2	127						
**P-values**									< 0.0001

**Fig. 2:**
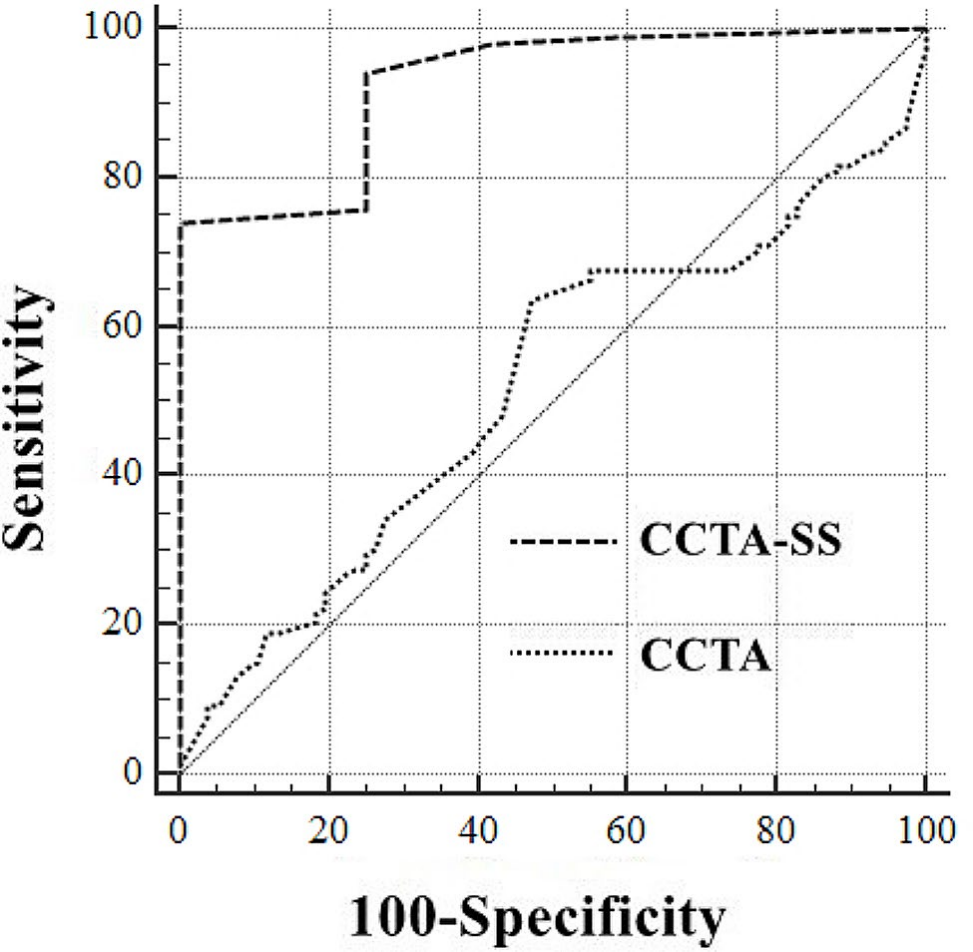
Receiver operating curve of coronary computed tomography angiography and coronary computed tomography angiography stenosis score for severe coronary artery disease diagnosis

For severe CAD, the AUC of the CCTA was 0.521 (95% CI: 0.454–0.587), whereas the AUC of CCTA-SS was 0.927 (95% CI: 0.885–0.957) (*P* < 0.0001) (Table 2; Fig. 2). The optimal cutoff points for CCTA and CCTA-SS were 68% and 51%, respectively. The sensitivity (CCTA-SS vs. CCTA: 92.70% vs. 75.91%), specificity (97.80% vs. 54.95%), positive predictive value (98.45% vs. 71.72%), negative predictive value (89.90% vs. 60.24%), and accuracy (94.74% vs. 67.54%), CCTA-SS were significantly higher than those of CCTA.

We further evaluated the 6-month revascularization of these severe patients. Among the 83 negative patients diagnosed with CCTA, 19 patients (22.89%, 19/83) underwent revascularization, and of 145 positive patients defined by CCTA, only 48 (33.10%, 48/145) received revascularization, accounting for 71.64% (48/67) of all revascularizations. For CCTA-SS, among the 99 negative patients diagnosed by CCTA-SS, only two patient (2.02%, 2/99) underwent revascularization after six months of follow-up; while in 129 positive patients, 65 (50.39%, 65/129) received revascularization, accounting for 97.01% (65/67) of all revascularizations.

## Discussion

This study evaluated the diagnostic performance of CCTA-SS for coronary stenosis that combined the data on plaque characteristics, myocardial functions, and diameter of stenosis. Using 228 patients with suspected CAD, CCTA-SS predicted CAD and severe CAD with more sensitivity, specificity, and accuracy than CCTA.

The incidence of CAD and CAD-related deaths are markedly increasing in the recent years. Thus, accurate and early prediction and evaluation of coronary stenosis are highly warranted for patients with cardiovascular diseases. In this study, we evaluated the effectiveness of CCTA-SS and demonstrated that it can provide a more accurate diagnosis of CAD and severe coronary stenosis than traditional CCTA.

Application of the calcification score decreases the disturbance of calcified plaques, especially diffuse calcification, which greatly increases the accuracy of diagnosing coronary artery disease [24, 25]. For patients with acute coronary syndrome, the pathological process involves three levels including the endothelium, myocardial function, and myocardial microcirculation [26]. CCTA-SS is a comprehensive method that combined plaque characteristics, the degree of stenosis of the subpericardium coronary lumen, early myocardial perfusion, and myocardial function of the segmental myocardium. Using both ≥ 50% and ≥ 70% of lumen stenosis from ICA as the gold standard, the accuracy of CCTA-SS in the diagnosis of CAD and severe CAD was significantly better than that of CCTA.

For CAD, if the true degree of stenosis is ≥ 70%, myocardial ischemia can occur even at rest [27], and the imaging features are visible as decreased myocardial perfusion or diastolic and systolic functions [28, 29]. When we used 50% and 70% of lumen stenosis diagnosed by ICA as the gold standard for diagnosis of CAD, there was a significant difference between CCTA and CCTA-SS in terms of CAD diagnosis. Therefore, CCTA-SS can significantly improve the early evaluation of ischemic-related CAD and the limits of CCTA were also corrected. Thus, CCTA-SS combined the characteristics of the plaque [30], early myocardial perfusion [31], and myocardial function [32] while effectively excluding interference factors for diagnosis, thus improving the accuracy of traditional CCTA.

This study has several limitations. First, the data was from a single center and the potential selection bias is unavoidable. Therefore, the CCTA-SS need to be verified in patients from across multiple centers in future. Second, misclassification bias might exist considering that all patients included were suspected to have coronary heart disease. Moreover, as we used ICA rather than invasive FFR as the gold standard, several patients may have been misclassified. Third, as this study is retrospective, it is unclear the extent to which that the treatments of patients (revascularization, conservative drug) would be influenced by the results of invasive coronary angiography. Moreover, the major adverse cardiac events in the two groups were not investigated and compared. At last, the enrolled patients didn’t include those relative special patients, such as young people aged ≤ 20 years, patients with long myocardial bridges, patients with obstructive pulmonary disease, and so on. Future well-designed prospective studies will be warranted to confirm the diagnostic performance of CCTA-SS in diagnosis and prediction for prognosis of CAD.

## Conclusions

In conclusion, CCTA-SS is a sensitive and specific method to diagnose CAD and severe CAD. The combination of plaque characteristics and myocardial function could significantly improve the diagnosis accuracy of coronary heart disease and provide an objective basis for clinical treatment.

### Electronic supplementary material

Below is the link to the electronic supplementary material.

**Supplementary Material 1**: Strategy of CCTA-SS scoring tool

## Data Availability

The datasets used and/or analysed during the current study are available from the corresponding author on reasonable request.

## Abbreviations

AUC: areas under curve
CAD: coronary artery disease
CCTA: coronary computed tomography angiography
CCTA-SS: coronary computed tomography angiography stenosis score
FFR: fractional flow reserve
GRACE: Global Registry of Acute Coronary Events
ICA: invasive coronary angiography
MPR: multi-planar reconstructions
MIP: maximum intensity projections
ROC: receiver operating curve
