# Supplementary material for: Diagnostic performance of coronary computed tomography angiography stenosis score for coronary stenosis
Source: BMC Med Imaging. 2024 Feb 9;24:39. doi: 10.1186/s12880-024-01213-8 (PMC10854174; doi:10.1186/s12880-024-01213-8)
Supplement: Supplementary file 1 — Supplementary Material 1: Strategy of CCTA-SS scoring tool [file 12880_2024_1213_MOESM1_ESM.pdf]

Table S1. Strategy of CCTA-SS scoring tool

**Features**		**Category**	**Score**
Plaque characteristics	Diffusion	Limit	3
		Segmental	5
		Diffuse	7
	Centrality	Eccentric	4
		Concentric	7
	Risk level	Calcifying	3
		Fibrous	5
		High-risk	7
Diameter of stenosis	Segment involvement score	< 50%	15
		50%～<70%	20
		70%～<90%	30
		90%～<95%	40
		≥ 95%	50
Myocardial functions	Early perfusion	Subendocardial	10
		Transmural	15
	Myocardial thickness	Local area	5
		Segmental area	10
	Thickening rate	Mild reduction	5
		Moderate reduction	10
		Severe reduction	15
